# Developmental Pathways Pervade Stem Cell Responses to Evolving Extracellular Matrices of 3D Bioprinted Microenvironments

**DOI:** 10.1155/2018/4809673

**Published:** 2018-03-29

**Authors:** Quyen A. Tran, Visar Ajeti, Brian T. Freeman, Paul J. Campagnola, Brenda M. Ogle

**Affiliations:** ^1^Department of Biomedical Engineering, University of Wisconsin, Madison, WI 53706, USA; ^2^Department of Biomedical Engineering, University of Minnesota-Twin Cities, Minneapolis, MN 55455, USA; ^3^Stem Cell Institute, University of Minnesota-Twin Cities, Minneapolis, MN 55455, USA; ^4^Lillehei Heart Institute, University of Minnesota-Twin Cities, Minneapolis, MN 55455, USA; ^5^Institute of Engineering in Medicine, University of Minnesota-Twin Cities, Minneapolis, MN 55455, USA

## Abstract

Developmental studies and 3D *in vitro* model systems show that the production and engagement of extracellular matrix (ECM) often precede stem cell differentiation. Yet, unclear is how the ECM triggers signaling events in sequence to accommodate multistep process characteristic of differentiation. Here, we employ transcriptome profiling and advanced imaging to delineate the specificity of ECM engagement to particular differentiation pathways and to determine whether specificity in this context is a function of long-term ECM remodeling. To this end, human mesenchymal stem cells (hMSCs) were cultured in 3D bioprinted prisms created from ECM proteins and associated controls. We found that exogenous ECM provided in 3D microenvironments at early time points impacts on the composition of microenvironments at later time points and that each evolving 3D microenvironment is uniquely poised to promote stem cell differentiation. Moreover, 2D cultures undergo minimal ECM remodeling and are ill-equipped to stimulate pathways associated with development.

## 1. Introduction

Although soluble factors supportive of differentiation of stem cells are well studied, our understanding of how extracellular matrix proteins (ECM) regulate differentiation is incomplete. Knowing the mechanistic contribution of the ECM to the dynamics of stem cell state is relevant for *in vitro* platforms for drug screening, toxicity testing, and disease modeling and is critical for *in vivo* therapeutic strategies involving tissue and whole-organ regeneration where ECM exposure is inevitable. A growing body of literature supports an association between exposure of stem cells to particular ECM types and specific differentiation outcomes. For example, Linsley et al. observed that hMSCs grown on two-dimensional type I collagen and fibronectin-coated surfaces differentiated towards the osteogenic lineage [1]. In addition, work by Lu et al. showed that acellular ECM generated by MSCs or chondrocytes was capable of inducing chondrogenic differentiation [2]. Similar studies have been extended to 3D environments [3, 4], where Jung et al. showed a complementary, but augmented, differentiation effect with 3D ECM exposure relative to that of 2D ECM [5, 6]. Similarly, Becerra-Bayona et al. examined mouse mesenchymal stem cell (mMSC) behavior in poly(ethylene glycol) (PEG) hydrogels conjugated with fibronectin, fibrinogen, and laminin and noted an increase in osteogenic differentiation in PEG hydrogels containing the latter two proteins [7]. Taken one step further, our lab has shown that ECM formulations can be optimized by using a “design of experiments” statistical approach to promote differentiation of particular cell types [8].

The impact of ECM on stem cell differentiation *in vitro* is perhaps not surprising given that *in vivo* developmental studies long ago demonstrated that the production and engagement of ECM often precedes differentiation events. For example, fibronectin has been shown essential for mesodermal, neuronal, and vascular development [9, 10]. Similarly, mass and clonal cultures of mouse cephalic and quail trunk neural crest were analyzed and it was found that fibronectin promotes differentiation of smooth muscle cells [11]. The effect was quite specific as differentiation of associated glia, neurons, and melanocytes was observed. In addition, the effect was not related to massive cell death or proliferation of smooth muscle cells [11]. But what is surprising is that most differentiation “programs” (whether of pluripotent or of multipotent cells) require multiple signals in sequence to achieve full maturation [12–14]. Does this mean ECM provides a first, middle, or end signal and requires additional soluble factor or cell-cell signaling to complete the sequence? *Or alternatively, can the ECM remodel or “evolve” to provide the stimulation sequence necessary for differentiation?* The latter scenario would require the stem cell or a supportive stromal cell type to institute the remodeling. This is important as the remodeling of the ECM and the subsequent change in cell activity have been shown to be important in processes such as vasculature and skeletal development, wound healing, and cancer development and progression [15, 16] as well as cell differentiation.

To determine whether the ECM evolves in association with differentiation, we devised an *in vitro* 3D model wherein multiphoton-excited (MPE) photochemistry was used to print 3D rectangular prisms composed of full-length type I collagen (Col1), fibronectin (FN), or laminin-111 (LN) proteins and containing human mesenchymal stem cells. Fabrication of the prisms occurs without addition of synthetic polymers, additional collagen type I, or other bioactive materials often added to support FN and LN which do not form spontaneous hydrogels ex vivo. The fabrication method is analogous to multiphoton laser scanning microscopy (MPLSM) in that the excitation, and thus, the photochemistry is restricted to the focal volume [17]. We demonstrated that MPE fabrication technology can crosslink soluble and structural proteins, layer by layer, into 3D protein matrices and fiber patterns with spatial fidelity of >85% [18]. We have characterized many of the material properties of the scaffold as well as examined stem cell-ECM interactions [19]. We have further shown that the cells adhere, migrate, and express focal adhesions on multiphoton excitation- (MPE-) crosslinked ECM scaffolds. Here, we used this 3D model system to study mechanistic underpinnings associating ECM engagement and remodeling with stem cell differentiation. (An earlier version of this work was presented as an abstract at the Biomedical Engineering Society Annual Meeting, 2017.)

## 2. Materials and Methods

### 2.1. Fabrication Instrument and Photochemistry

The multiphoton fabrication instrument has been described in detail previously and is only described here briefly [18]. A ti:sapphire femtosecond laser is coupled to an upright microscope stand (Axioskop 2, Zeiss, Thornewood, NY), and scanning is performed through a combination of laser scanning galvos (Cambridge Technologies, Bedford, MA) and a motorized stage (x-y-z, Ludl Electronic Products Ltd., Hawthorne, NY) under LabVIEW control with a field-programmable gate array (FPGA) board (Virtex-II PCI-7831R, National Instruments, Austin, TX) functioning as a data acquisition element (DAQ) [18]. Fabrication parameters such as power, scanning area, scan rate of galvos, and repetition of scanning pattern (#scans/layer) are set within the graphical user interface (GUI).

An FPGA was incorporated in the fabrication system to exploit parallelism of command executions (80 MHz clock rate) and to avoid bottlenecks in communications between the central processing unit (CPU) and hardware through four of the first-in, first-out (FIFO) channels. The first two FIFO channels relay information from the main LabVIEW program to the FPGA to control the galvo mirrors and fast electrooptic modulator (EOM) shutter, while the other two record information from the photomultiplier tube (PMT) to create a live image of the fabrication making the communication between the CPU and hardware near real time. The source code of the instrument control software is freely available at: http://campagnola.molbio.wisc.edu/.

The two-photon excitation of the Rose Bengal photoactivator is induced by a femtosecond titanium sapphire laser (Mira, Coherent, Santa Clara, CA) operating at 780 nm. The photochemistry proceeds through the generation of singlet oxygen which then attacks residues containing aromatic groups and free amines [20]. The resulting radical protein then links to a second protein molecule, generating a covalent bond. A 20x, 0.75 numerical aperture objective lens was used.

### 2.2. Structure Fabrication

Three-dimensional scaffolds were fabricated from solutions containing pure BSA (Sigma-Aldrich, St. Louis, MO), BSA and murine laminin-111 (LN) (isolated from Engelbreth-Holm-Swarm mouse sarcoma, EMD Millipore, Darmstadt, Germany), BSA and fibronectin (FN) (isolated from bovine plasma, Sigma), or BSA and collagen type I (Col1) (isolated via acetic acid digest from rat tail, Sigma), where the concentrations of BSA was 50 mg/mL for all scaffolds and for scaffolds with ECM added, the ECM concentration was 0.5 mg/mL for each individual ECM protein. BSA was used alone as a negative control for ECM exposure and also in the samples containing ECM protein, to provide enhanced structural stability [19]. Scaffold dimensions were set at 350 × 350 × 100 *μ*m to ensure the complete encapsulation of MSCs and to maintain structure integrity.

Scaffolds were linked to a nonspecific BSA self-assembled monolayer (SAM) linked to another organosilane SAM on a glass slide. The slides were prepared by (i) plasma cleaning, immersion in octadecyltrichlorosilane (OTDS) (Gelest Inc., Morrisville, PA), (ii) washing with anhydrous toluene to remove any residual ODTS, (iii) drying with N_2_, and (iv) heating for 30 min at 120°C to complete the formation of the Si^−^O bonds of the self-assembled organosilane monolayer. The silanized slides were then soaked in a 10 mg/mL solution of BSA to form the background self-assembled monolayer and then rinsed.

The protein solution and Rose Bengal photoactivator (2 mM) were confined in a small circular rubber chamber (Grace Bio-Labs, SA8R-0.5) seated on top of the BSA monolayer. Fabrication parameters such as structure size, laser power, size of axial steps, and scanning rate were optimized for maximum crosslinking without photodamage. After fabrication, scaffolds were exposed to high laser power to photobleach the residual Rose Bengal to ensure cell viability with prolonged imaging. Slides were then immersed in 1X PBS pH 7.4 (GIBCO) containing 400 *μ*g/mL penicillin and 400 *μ*g/mL streptomycin under sterile conditions and kept hydrated for cell plating.

### 2.3. Fluorescence Lifetime Imaging Microscopy

Fluorescence lifetime imaging microscopy (FLIM) was used to image MSCs inside the 3D structures where the respective contrasts were DAPI staining of the cell nucleus and residual entrapped Rose Bengal. The FLIM images were acquired on a custom-built, multiphoton microscope located at the Laboratory for Optical and Computational Instrumentation (LOCI) using time-correlated single photon counting (TCSPC; SPC830, Becker and Hickl, Berlin, Germany). Images were taken with a 40 Å~1.15 NA water immersion objective, where each optical section had a field of 512 Å~512 pixels and 64 time bins per pixel and required 60 s for acquisition. A z stack was comprised of 100 optical sections with a 1 *μ*m axial step size. All FLIM measurements used two-photon excitation at 890 nm, and the DAPI emission was collected with a 520/35 nm filter (Chroma Technology, Rockingham, VT), whereas Rose Bengal fluorescence was collected with a 620/35 nm filter (Chroma Technology). FLIM images were fitted using the hardware-bundled analysis software (SPCImage, Becker-Hickl) to a single exponential decay model. Analyzed images were color mapped according to the fluorescence lifetime, exported, and reconstructed in 3D using Imaris software (Bitplane, Zurich, Switzerland).

### 2.4. Physical Characterization of BSA, BSA/FN, BSA/Col1, and BSA/LN Scaffolds

#### 2.4.1. Determination of Volumetric Swelling Ratio

To characterize the relative crosslinking of the structure, the volumetric swelling ratios were measured, where this is defined as the ratio of the hydrated to the dehydrated volume [19]. The former was determined by obtaining two photon-excited fluorescence (TPEF) images (890 nm excitation) of the structure in physiologic medium at 1 *μ*m axial step sizes with an 0.8 NA objective lens, where the contrast was from residual entrapped Rose Bengal. The structures were dehydrated by immersion in 100% ethanol and then dried completely and imaged under the same conditions as the hydrated case. The areas and heights (and resulting volumes) were determined using the freely available FIJI image analysis software (http://fiji.sc/wiki/index.php/Fiji).

### 2.5. Fractal Dimension Determination

Scanning electron microscopy (SEM) was used to determine the structural assembly at higher resolution than by optical microscopy, where we specifically determine the fractal dimension, rather than pore sizes or their distribution. Prior to scanning, structures were fixed overnight (4°C) using a 0.1 M phosphate buffer containing 1.5% glutaraldehyde and 1% tannic acid and then dehydrated using a series of ethanol washes and a critical point drying step (Samdri 780 critical point drier, Tousimis, Research Corp., Rockville, MD). Lastly, gold/palladium (60 : 40) was deposited onto the structures, with a thickness of 30 nm, using a DC sputter coater (Auto Conductavac IV, Seevac Inc., Pittsburgh, PA). SEM images were acquired using the Hitachi S-570 microscope (Hitachi, Tokyo, Japan). The mean fractal dimension was computed using the FIJI FracLac plugin.

### 2.6. Cell Culture

Embryonic stem cell-derived mesenchymal stem cells [21, 22] were cultured in *α*MEM, 10% fetal bovine serum, 1% L-glutamine, 1% nonessential amino acid, and 1% penicillin/streptomycin. hMSCs were plated at 10,000 cells/mm^2^ with daily media changes for 28 days. hMSCs used for scaffold modification analysis were seeded at a concentration of 5000 cells/cm^2^ and cultured for 14 days. hMSCs used for MMP, integrin, and ECM analysis were seeded at 10,000 cells/cm^2^ and cultured for 28 days.

### 2.7. Tracking of BSA, BSA/FN, BSA/FN, and BSA/LN Physical Structure Modifications

To track physical modification of the structures, phase contrast images of the structures were taken daily for 14 days. Images were obtained on an Axiovert 40C microscope (Zeiss) using a 10x objective with a 0.25 NA. Structure size was measured using the freely available Fiji software (http://fiji.sc/wiki/index.php/Fiji, Supplemental Table 1).

### 2.8. Gene Expression Analysis Using RNA Sequencing

hMSCs specifically within the structures were excised and RNA was extracted according to the RNeasy Mini Kit (Qiagen, Germantown, MD). cDNA was generated using the SMARTer Ultra Low RNA kit (Clontech, Mountain View, CA). mRNA library was produced according to the Illumina Nextera XT preparation kit's manufacture protocol (Illumina, San Diego, CA). RNA was sequenced using the Illumina MiSeq sequencher with paired end reads, a length of 75 bp, and a depth 20 million reads. This work, except RNA extraction, was performed at the University of Minnesota Genomics Center.

Gene expression was analyzed using the Galaxy software (Minnesota Supercomputing Institute (MSI), University of Minnesota, MN), and all generated data can be found on the GEO database, accession number GSE102737, reviewer token OGLE30. RNA sequencing reads were aligned to the human genome (hg19.fa and hg19_genes_2012–03-09.gtf) using the TopHat software (version 2.0.09, open source software, http://ccb.jhu.edu/software/tophat/index.shtml). TopHat results were further analyzed using Cufflinks (version 2.2.1, open source software, http://cole-trapnell-lab.github.io/cufflinks/) software to assemble the gene transcripts and estimate gene abundance. Read counts were normalized to obtain FPKM (fragment per kilobase of transcript per million mapped reads, Supplemental Table 1). Differential gene expression was determined using the single-cell differential expression (SCDE) toolset [23, 24]. Genes with a *q* value less than 0.05 were considered “differentially expressed.” Gene-annotation enrichment analysis was performed with the Database for Annotation, Visualization and Integrated Discovery (DAVID) informatics resources 6.7 of the National Institute of Allergy and Infectious Diseases (NIAID) and of the National Institutes of Health (NIH) (Supplemental Tables 3 and 4).

### 2.9. Quantitative Polymerase Chain Reaction

The cDNA for RNA sequencing verification was amplified from cDNA used for RNA sequencing. Primers for COL1, SGPL1, and MAGED2 were purchased from Qiagen, and GAPDH (forward: TTAAAAGCAGCCCTGGTGAC reverse: CTCTGCTCCTCCTGTTCGAC) was used as an internal control. Quantitative polymerase chain reaction (PCR) was performed using SYBR Green Master Reagent (Thermo Fisher Scientific, Waltham, MA) and ran on an Applied Biosystems StepOne Plus machine. Gene fold change was determined using the ΔΔ*C*_t_ method with each gene normalized to GAPDH and 3D/FN normalized to BSA structures.

### 2.10. Statistical Analysis

Volumetric swelling ratio, mean fractal dimension, and changes in ECM, integrin, and MMP expression were analyzed for statistical significance using ANOVA with Tukey post hoc analyses using JMP software (SAS, Cary, NC). Hierarchical clustering and principal component analyses were conducted using R software.

## 3. Results

### 3.1. Physical Characterization of 3D ECM-Based Bioprinted Prisms

**Figure 1:**
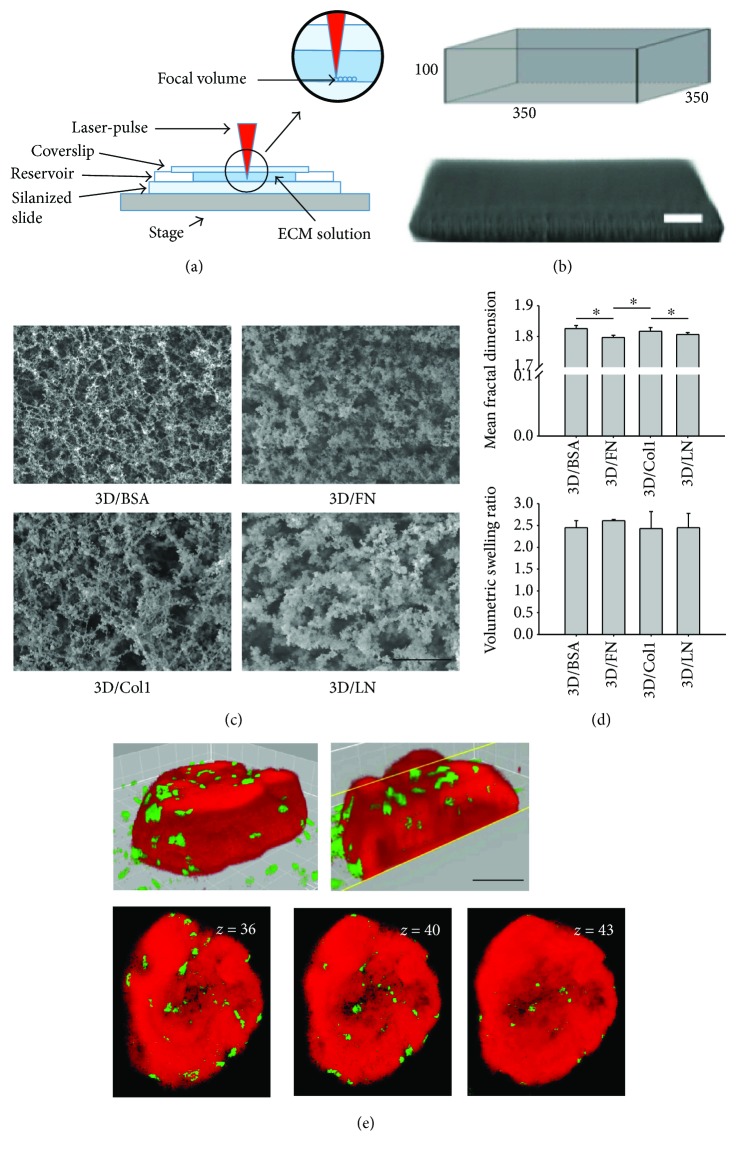
Fabrication of 3D ECM-based, bioprinted prisms. (a) Fabrication Schematic. Multiphoton excitation was used to polymerize a focal volume containing individual ECM proteins (e.g., FN, Col1, and LN) and associated photocrosslinking agent. 3D printing of this type was used so that a three-dimensional construct, in this case a rectangular prism, could be generated even with ECM types that do not form hydrogels spontaneously ex vivo. (b) Geometric template (above; dimensions of micron scale) and associated bioprinted ECM-based rectangular prism containing BSA and LN (BSA/LN; below). Scale bar = 50 *μ*m. (c) Representative SEM images of prisms fabricated with BSA, FN, Col1, and LN. Scale bar = 10 *μ*m. (d) Average fractal dimension of each ECM-based, 3D bioprinted prism (above); volumetric swelling ratio of each ECM-based bioprinted prism (below). Error bars depict standard deviation (SD), ^∗^ *P* < 0.05, *n* = 3 experimental replicates. (e) Multiphoton imaging to show interaction of hMSCs with bioprinted prism containing BSA only after 3 days of seeding. 3D reconstruction (left) and cut-away view (right) show cellular infiltration (e, upper panels). Also, in support of hMSC infiltration are shown multiple cross sections at various *z* depths (e, lower panels). Green (CD90) indicates MSCs; red indicates the bioprinted matrix. Scale bar = 100 *μ*m.

To track the interplay between 3D ECM exposure and hMSC differentiation, we selected ECM proteins for our 3D *in vitro* model representing the primary classes of ECM found in stromal environments, namely, fibrillar collagens, basement membrane, and small adaptor proteins. Thus, 3D bioprinted prisms were fabricated from ECM protein collagen type I (Col1), laminin-111 (LN), or fibronectin (FN) supplemented with bovine serum albumin (BSA) to improve crosslinking efficiency and subsequently seeded with hMSCs. 3D bioprinting was accomplished via multiphoton excitation-based fabrication and was utilized here so that hMSCs could be exposed to ECM proteins in 3D, even ECM proteins that do not spontaneously form hydrogels outside the body (Figures 1(a) and 1(b)). Bioprinted prisms containing different ECM proteins were physically characterized in terms of pore size and relative crosslinking density. We found that the ECM type slightly affected the topography of the structure in terms of fractal dimension but the associated crosslinking density remained consistent between structures (Figures 1(c) and 1(d)). For this reason, altered differentiation outcomes following culture in the 3D bioprinted prisms will largely reflect biochemical differences of the prisms, but also nuances in topography. hMSCs were seeded on 3D bioprinted prisms, and soon after seeding, hMSCs infiltrated the prisms and maintained viability in the prisms for several weeks [19] (Figure 1(e)).

### 3.2. Transcriptional Profile of hMSCs in 3D ECM-Based Bioprinted Prisms

**Figure 2:**
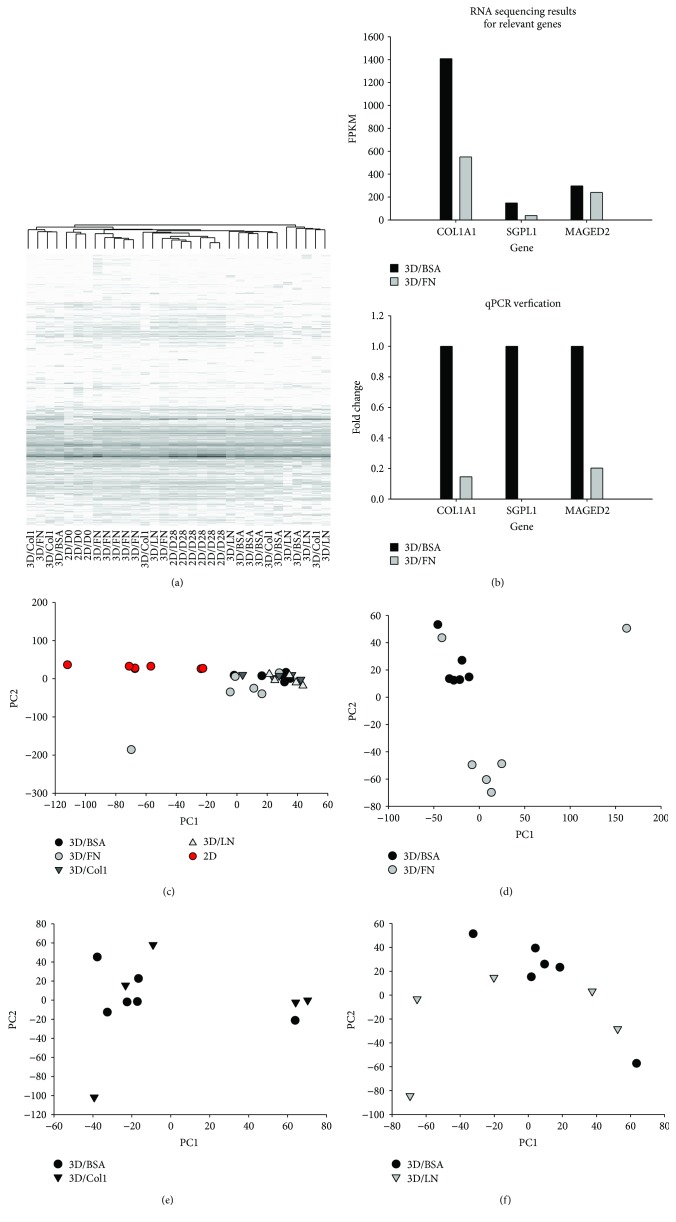
RNA sequencing analysis of hMSCs in 3D bioprinted prisms at 0 and 28 days. (a) Hierarchical clustering of all genes with FPKM > 1 for 3D bioprinted prisms containing either BSA alone (3D/BSA), 3D/FN, 3D/Col1, or 3D/LN at day 28 and associated 2D control cultures at day 28. (b) qPCR validation of gene expression of a subset of genes. These genes were selected as they represent families of ECM proteins and differentiation markers associated with hMSC progeny. (c) PCA analysis of all genes with FPKM > 1. (d–f) Comparison of individual 3D bioprinted prisms containing ECM to bioprinted prisms with BSA only.

Given the myriad of differentiation outcomes of hMSCs, we decided to employ RNAseq of hMSCs to ensure global assessment of transcriptional outcomes of ECM exposure in our 3D *in vitro* model. Thus, after 28 days of culture, RNAseq was conducted on hMSCs of 3D bioprinted prisms (termed 3D/Col1, 3D/LN, and 3D/FN) as well as controls including hMSCs just prior to matrix seeding (2D/D0), hMSCs cultured for 28 days on tissue culture polystyrene (2D/D28), and hMSCs infiltrating 3D printed prisms composed of bovine serum albumin (3D/BSA) for 28 days. 3D/BSA was used to control for BSA added for structural stability of the bioprinted prisms with ECM and to distinguish outcomes associated with engagement of the integrin family of receptors since BSA does not bind integrins. Overall gene expression profiles were analyzed via hierarchical clustering and principal component analysis (PCA) to determine the extent of similarity/dissimilarity between experimental groups based on overall mRNA expression profiles for each sample. Hierarchical clustering group samples were based on mRNA expression profiles over a variety of scales by creating a cluster tree or dendogram where clusters of samples at one level are joined as clusters at the next level, allowing one of determine the scale of clustering or association for the cell populations exposed to disparate ECM in 3D. PCA uses an orthogonal transformation to convert the set of mRNA expression data for each sample that may be correlated into a set of linearly uncorrelated variables called principal components. The transformation is defined such that the first principal component represents the largest possible variance and the second principal component has the highest variance possible under the constraint that it is orthogonal to principal component 1. The resulting vectors are an uncorrelated orthogonal basis set which, when plotted on an *x*-*y* grid, can reveal unbiased associations between mRNA expression levels of two or multiple samples (in this case, cell populations exposed to disparate ECM in 3D scaffolds) based on proximity on the plot. The hierarchical clustering and PCA analyses included all genes of each sample with an FPKM value greater than 1. Analysis showed that the initial hMSC population (2D/D0, *n* = 3 populations from three independent experiments, but same passage) and hMSCs after 28 days in standard 2D culture (2D/D28, *n* = 6 populations from three independent experiments, but same passage) cluster far from each other (Figures 2(a) and 2(c)) suggesting that the extended duration in culture without passaging alters the transcriptome, which is consistent with previous reports [25]. hMSCs cultured in 3D bioprinted prisms (*n* > 5 prisms for each ECM type from three independent experiments, but same passage) cluster away from those in 2D at 28 days, suggesting that the transition from 2D to 3D culture also has a substantial effect on the hMSC transcriptome. Clustering differences between 3D prisms of different ECM composition are more subtle and easier to visualize when compared, independent of the 2D controls. When viewed in this way, we observed a separation between 3D/BSA and 3D/LN or 3D/FN indicating that the presence of FN and LN substantially altered the gene expression of hMSCs after 28 days of 3D culture (Figures 2(d)–2(f)). However, PCA analysis revealed that 3D/Col1 structures did not vary from BSA, suggesting that provision of exogenous, full-length collagen type I did not augment or change hMSC transcript expression relative to the 3D albumin base. RNA sequencing data was verified using quantitative PCR, and indeed, the trend in the FPKM levels of COL1A1 (alpha 1 chain of type I collagen), SGPL1 (sphingosine-1-phosphate lyase 1), and MAGED2 (MAGE family member D2) genes matched quantitative PCR results (Figure 2(b)). Thus, gene expression profiles of hMSCs between 2D standard culture and 3D structures vary substantially, with significant but more subtle differences emerging between 3D structures fabricated from distinct ECM protein types.

### 3.3. Protein Degradation, Differentiation, and Development Pathways Altered in Association with Specific ECM Proteins

Gene ontology (GO) analysis was performed on differentially expressed genes to identify pathways significantly altered by 3D culture and varied exogenous ECM of 3D prisms. The two pathways most significantly altered by 3D culture were (1) protein degradation and (2) development and differentiation (Supplementary Table 2). These processes even exceeded proliferation, migration, and cytoskeletal activation, indicating that the environment generated at this time point (28 days) was conducive to matrix remodeling and cell specification. We therefore began by exploring the effect that the cells exerted to remodel the 3D bioprinted environments.

### 3.4. Quantification of Matrix Remodeling

**Figure 3:**
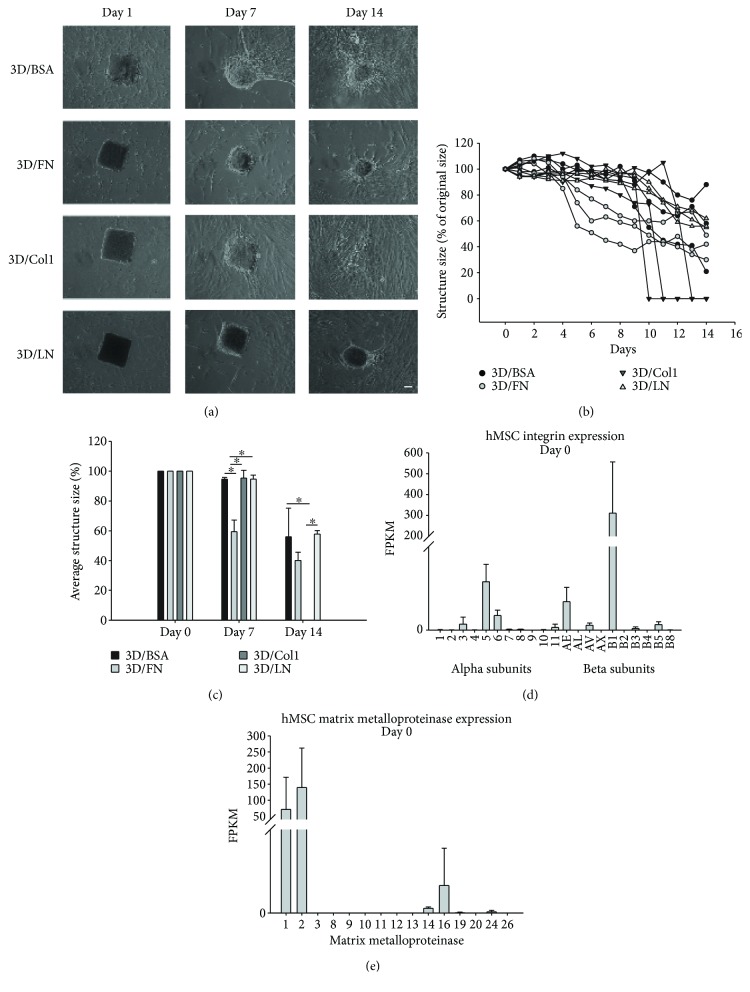
Physical modification of three-dimensional structures composed of 3D/BSA, 3D/FN, 3D/Col1, and 3D/LN by hMSCs. (a) Representative phase contrast images of 3D/BSA, 3D/FN, 3D/Col1, and 3D/LN structures on day 1, 7, and 14. Scale bar = 50 *μ*m. (b) Line graph showing changes in 3D/BSA, 3D/FN, 3D/Col1, and 3D/LN structure size over a two-week period. (c) Bar graph showing the average structure size after 14 days in culture. Error bars depict SD associated with *n* = 2 experimental replicates with at least 3 different prisms analyzed per experimental replicate. ^∗^ *P* < 0.05. (d, e) Expression levels of integrins and matrix metalloproteinases in day 0 hMSC population. (d) Bar graph showing the expression of *α*3, *α*5, *α*6, *α*11, *α*E, *α*V, *β*1, *β*3, and *β*5 integrin subunits in day 0 cells. (e) Bar graph showing the expression of MMP1, 2, 14, 16, 19, and 24 in day 0 hMSCs.

In order to understand how hMSCs remodel their external environment, we initially examined the physical modifications that the cells exerted on the 3D bioprinted prisms. hMSCs were introduced to 3D/BSA, 3D/FN, 3D/Col1, and 3D/LN prisms and cultured for two weeks. Physical modifications to the prisms were tracked by examining changes in structure for the duration of the experiment. Representative phase contrast microscope images for the four different prisms at days 1, 7, and 14 are shown in Figure 3(a), and the quantitative temporal evolution is shown in Figures 3(b) and 3(c). hMSCs associated with BSA prisms migrated into and around the entire structure without large modifications to the structure during the first week. The second week of culture resulted in reduced matrix size to varying degrees (loss of 10–80%). The cells of the BSA/FN prisms rapidly modified the structure, which continued to decrease in size during the 2-week duration. Cells of the BSA/Col1 prisms did not greatly alter the structure dimensions in the first week. However, the cells rapidly degraded the prisms during the second week resulting in complete destruction of the structures. BSA/LN structures retained the original features in the first week but were reduced in size by the hMSCs during the second week. The final size of the structures relative to the initial condition is shown in Figures 3(b) and 3(c). Overall, hMSCs physically manipulated the structures with varying kinetic, and to different degrees, the order is from highest to lowest in terms of final size: 3D/Col1 < 3D/FN < 3D/BSA < 3D/LN.

### 3.5. Kinetics of Integrin, Matrix Metalloproteinase, and Extracellular Matrix Expression in Bioprinted Prisms

The varying kinetics and degree of modification to the prisms may reflect the initial state of the hMSCs, particularly the expression of integrin family members. Integrins are potent ECM adhesion and contraction molecules where each family member harbors specificity for particular ECM proteins. Thus, we examined the initial expression level of the common *α* and *β* subunits by hMSCs via the RNAseq data (Figure 3(d)). We found that MSCs expressed the integrin *α* and *β* subunits associated with fibronectin (*α*5, *α*V, and *β*1), laminin (*α*3, *α*6, and *β*1), and collagen type I (*α*11, *β*1) [26, 27]. However, these integrin subunits were detected at different levels and can be ranked from highest to lowest according to ECM affinity as follows: FN > LN > collagen type I (Figure 3(d)). This level of expression complements the kinetics of remodeling such that those prisms with high levels of integrins with binding capacity (3D/FN) were restructured more quickly than those with lower levels of integrins with binding capacity. This could also account for the mild and sporadic degradation of the 3D/BSA prisms, which lack a direct interface to the powerful actin cytoskeleton afforded by integrin engagement.

The integrins typically associated with Col1 binding (*α*1*β*1 and *α*2*β*1) were not well expressed by the hMSCs, which may account for the lag in remodeling kinetics. However, the 3D/Col1 structures are completely degraded at 2 weeks. Therefore, we examined the initial expression of matrix metalloproteinases (MMPs), enzymes that degrade specific ECM proteins dependent on the MMP family member. We observed highest expression of MMP1 and MMP2, which are both capable of collagen type I degradation (Figure 3(e)). Thus, the initial expression of these specific MMPs may have allowed MSCs to slowly but substantially degrade the 3D/Col1 structures. Notably, MMP1 does not significantly degrade FN [28] or LN [29], while MMP2 can degrade FN, but not LN. Of the other four MMPs expressed at moderate levels in hMSCs at day 0 (MMP14, MMP16, MMP19, and MMP24), only MMP14 can degrade collagen type I, FN, and LN [30–32]. Taken together, the speed with which ECM prisms were remodeled reflects initial integrin expression, while the extent of degradation is likely tied more closely with MMP expression.

Since complete degradation of prisms appeared to accelerate in the second week for all prisms except 3D/FN, we examined changes in MSC genes associated with matrix remodeling, including endogenous ECM (Table 1), integrins (Table 2), and MMPs (Table 3), *after 28* days with a focus on those genes significantly altered between prism type. Interestingly, we note that expression levels of most integrins were not significantly altered between most 3D conditions and relative to 2D controls (Table 2, Figure 3(d)). We next examined differential expression of MMPs between 3D bioprinted prisms at day 28 (Table 3). MMP16 was upregulated in 3D/Col1 bioprinted prisms. MMP16 is a membrane-type metalloproteinase shown to cleave and thereby activate MMP2, which is known to cleave collagens. In addition, MMP13 was upregulated in 3D/Col1 and is known to degrade type I collagen, though with a preference for type II collagen. Augmented expression of MMP13 and MMP16, together with sustained expression of MMP1 and MMP2, supports the rapid and complete degradation of 3D/Col1 prisms in week 2. The incomplete degradation of 3D/FN and 3D/LN structures may reflect the low or lack of expression of MMPs specific for these ECM proteins, namely, MMP7, MMP10, MMP11, MMP14, and MMP15 for LN and MMP7, MMP10, MMP11, MMP12, MMP14, and MMP15 for FN. In addition, cells cultured in 3D/LN exhibited high levels of tissue inhibitor of metalloproteinases (TIMP) 3.

Ignored in the analyses thus far are newly deposited ECM proteins that likely to also contribute to remodeling kinetics of ECM prisms. Table 1 shows most dramatic changes in transcript expression of fibronectin, syndecan4, versican, and tenascin C between ECM prism types. Of course, transcript expression is not necessarily indicative of deposition and this snapshot is sure to miss ECM that may have been deposited at intermediate time points. However, differential expression of ECM between prisms further supports the premise that exposure to different ECM proteins at early time points results in the specific evolution of microenvironments supportive of distinct behaviors.

### 3.6. Development and Differentiation Behaviors Prevalent in Evolving ECM Environments

**Figure 4:**
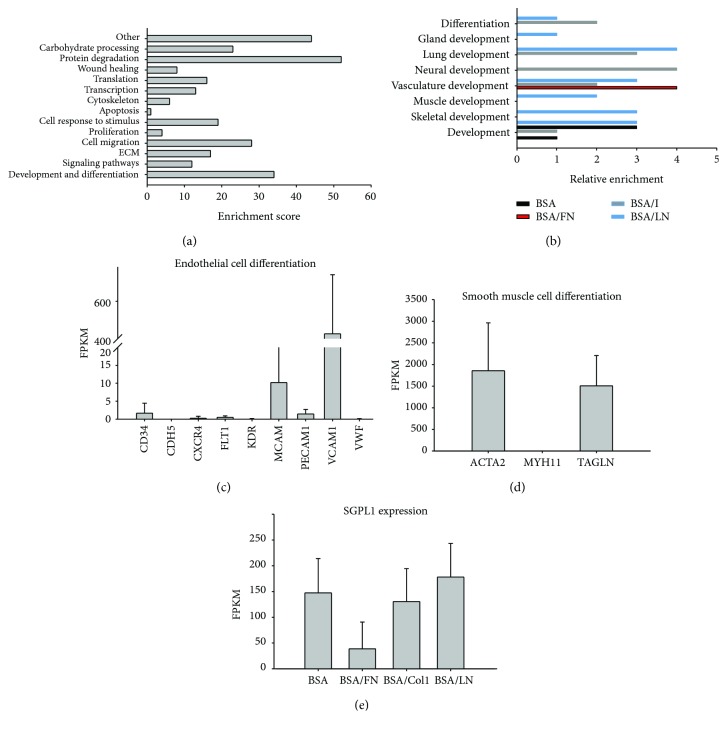
GO analysis of hMSCs in 3D bioprinted prisms, especially related to the differentiation state. (a) Counts of GO terms associated with commonly examined stem cell responses. Protein degradation, development, and differentiation are the most significantly expressed pathways. (b) Development and differentiation pathways separated by 3D ECM structures. Most influential 3D structures rank from 3D/LN > 3D/Col1 > 3D/BSA > 3D/FN, with 3D/FN structures predominantly influencing vasculature development. Expression of endothelial cell markers (c), smooth muscle cell markers (d), and sphingosine phosphate lyase (e) in hMSCs cultured in 3D/FN structures. Error bars depict SD associated with *n* = 3 experimental replicates with at least 2 different prisms analyzed per experimental replicate. hMSCs cultured in 3D/FN structures expressed markers for both endothelial and smooth muscle cells and had a reduction in SGPL1 expression.

Development and differentiation pathways were also prevalent in cells of 3D prisms, even exceeding proliferation, migration, and cytoskeletal activation, indicating that the environment generated at this time point (28 days) was conducive to cell specification. Upon closer examination of the development and differentiation pathways, we noted that a larger number of developmental pathways compared to differentiation pathways were significantly affected. Interestingly, many of these developmental pathways are not typically associated with MSCs, such as lung and gland development. In addition, the developmental or differentiation pathways triggered were ECM specific in some cases (Figures 4(a) and 4(b)). In particular, skeletal development was associated with 3D/BSA and 3D/LN structures; muscle development and gland development were altered by 3D/LN structures; neural development was linked with 3D/Col1 structures; lung development and overall differentiation pathways were associated with 3D/LN and 3D/Col1 structures. Vasculature development was connected to all ECM-based prisms with 3D/FN structures well-surpassing the others. Interestingly, 3D/FN was only associated with vasculature development, indicating the utility of this ECM protein in 3D tissue engineering efforts that seek to include a functioning vasculature.

### 3.7. Vasculature and Blood Vessel Development in 3D/FN Structures

We further probed vascular development in the 3D/FN structures given the need in the tissue engineering field to generate functioning and integrated vascular networks for thick tissues. In particular, we inspected the expression of key endothelial and smooth muscle cell markers, as these cells are important in the development of a functioning vasculature network. hMSCs cultured in 3D/FN structures for 28 days expressed both endothelial cell (MCAM, VCAM) and smooth muscle cell markers (ACTA2, TAGLN) indicating that exogenous, full-length fibronectin protein presented in 3D can trigger differentiation of hMSCs into these two cell types (Figures 4(c) and 4(d)). We also probed for spingosine-1-phosphate (S1P), a sphingolipid that has been shown to play a role in vasculature development and is irreversibly degraded by SGPL1 [33–35]. We noted a significant reduction in sphingosine phosphate lyase (SGPL1) in cells cultured in 3D/FN structures specifically (Figure 4(e)). Thus, while functional maturation of vascular cell types in 3D/FN remains to be seen, initiation of these differentiation pathways is strongly supported.

## 4. Discussion

In this work, we examined the global influence on transcription of full-length type I collagen, fibronectin, and laminin-111 individually on hMSC behavior after 28 days of 3D culture. Predominant outcomes reflected changes in protein degradation, differentiation, and development pathways. Interestingly, we observed no statistical difference in the differentiation of MSCs towards the adipogenic and chondrogenic lineages; however, alternate developmental pathways including lung, neural, vascular, and muscle development were activated and such activation was related to the original composition of the 3D ECM-based microenvironment. The ECM composition of the microenvironments changed substantially during the four-week-study duration supporting the notion that evolving ECM may provide temporal cues required for differentiation to multiple different lineages.

Among the most striking differentiation outcomes was vascular differentiation associated with fibronectin. The differentiation of stem cells into endothelial cells in connection with fibronectin has previously been demonstrated [36]. In particular, studies by Battista et al. examined the differentiation of mESCs in the presence of LN or FN in a collagen I hydrogel and observed that fibronectin stimulated EC differentiation and vascularization while LN stimulated cardiomyogenic differentiation [37]. Additionally, studies like those by Clark et al. indicated a close association between fibronectin and endothelial cell activity [38]. But this is the first study to show that the dominant response of MSCs to fibronectin exposure is vascular differentiation and it is one of the most potent ECM-stimulated responses that we observed in the context of our 3D model system. It should be noted that our 3D model system includes a crosslinking regime that could hide critical cell binding or soluble factor sequestration sites and thereby complicate comparison to native tissue or other 3D model systems that do not include exogenous crosslinking of this type. Even so, these studies suggest inclusion of full-length fibronectin in engineered tissue, especially that thick tissues with a vascular requirement may be advantageous.

As evidence builds supporting the notion that the extracellular matrix is a potent signaling molecule, it is now time to address what happens following integrin engagement and before transcription of proteins associated with a maturing cell type. The design of this study was not crafted to be able to address this question effectively since we can only access beginning and end events in the kinetics of pathway activation with ECM-guided differentiation. Future studies might benefit from an altered design wherein signaling dynamics could be captured. There are a few recent publications that begin to probe these dynamics. For example, peptide activation of *α*5*β*1 can drive osteogenic differentiation of mesenchymal stem cells via the Wnt/*β*-catenin pathway activated via PI3K/Akt signaling [39]. In addition, engagement of fibroblast-derived ECM via *β*1, *α*2, and *α*3 integrins in human embryonic stem cells has been shown to activate the Wnt/*β*-catenin pathway via the MEK-ERK pathway, which drives endoderm differentiation [40]. Finally, we have preliminary evidence to suggest that activation of integrin-linked kinase (ILK) of focal adhesions couples *β*-catenin activation via GSK3*β* to enable cardiomyocyte differentiation. Missing in these studies is consideration of the multistep process inherent in any differentiation outcome. Thus, while provision of exogenous ECM might provide a potent “signal 1,” the source of subsequent signals is unknown and could arise from stimulation of endogenous ECM, degradation of ECM, soluble factor synthesis, or soluble factor sequestration. Discerning the source of signals 2, 3, and so on for ECM-guided differentiation and manipulation of associated intracellular signaling pathways like those described above will be useful in the context of ECM-based *in vitro* platforms for drug screening, toxicity testing, and disease modeling and will *be critical* for stem cell-based therapeutic strategies where ECM exposure is inevitable.

## Figures and Tables

**Table 1:** Expression levels of ECM proteins in hMSCs cultured for 28 days in 2D standard and 3D ECM-based structure conditions.

Gene	Average FPKM					Significance
	2D/D28	3D/BSA	3D/FN	3D/Col1	3D/LN	
COL1A1	1694.69	**1408.85**	**550.58**	*1796.03*	**656.82**	
COL1A2	1437.28	**967.59**	**596.87**	**1363.82**	**1391.45**	
COL3A1	331.77	*582.23*	*363.92*	*549.25*	*694.15*	
COL4A1	39.19	**16.04**	**16.18**	**27.23**	**3.37**	
COL4A5	3.16	*8.90*	*8.37*	*10.34*	0.00	
COL5A1	34.22	**27.83**	**21.03**	**20.68**	**15.59**	
COL5A2	30.84	*42.84*	**29.80**	*55.98*	**28.19**	
COL6A1	22.61	*68.66*	*72.65*	*61.24*	*47.19*	
COL6A3	102.42	*196.03*	*139.69*	*173.26*	*177.62*	
COL8A1	24.32	*52.07*	*61.73*	*76.64*	*100.51*	
COL8A2	7.88	*12.63*	**5.02**	*8.64*	**2.20**	
COL10A1	5.34	*6.97*	**4.53**	*9.20*	*11.18*	
COL11A1	10.60	*58.77*	*16.37*	*59.43*	*39.83*	
COL12A1	3.44	*13.00*	*8.38*	*11.41*	*10.26*	
COL14A1	0.53	*4.33*	*10.65*	*7.61*	*6.84*	
FN1	2206.39	*3823.51*	**2129.52**	*4017.30*	*5628.25*	d, h
LAMA4	15.13	*36.67*	*30.44*	*30.82*	*46.42*	
LAMB1	67.78	*107.45*	**65.75**	111.18	**67.06**	
LAMB2	11.67	**4.08**	**7.36**	**8.31**	*14.50*	
LAMC1	28.32	*65.12*	*31.50*	*61.57*	*39.43*	
LAMC2	7.05	*11.45*	*9.53*	*15.77*	**0.33**	
CD44	96.64	*120.43*	*132.88*	*179.45*	*168.33*	
ELN	16.18	**2.01**	0.97	**1.65**	0.15	
FBN1	30.03	*84.49*	*54.72*	*85.81*	*86.47*	
FBLN1	80.54	*170.48*	*126.57*	*131.39*	*215.67*	
FBLN5	95.26	*118.34*	**52.77**	**75.25**	**77.58**	
SDC2	16.83	**14.14**	**13.80**	**12.82**	**12.50**	
SDC4	18.49	**2.18**	**11.96**	**8.78**	**5.02**	a, c, d, f
TNC	38.44	*179.79*	*83.47*	*168.76*	*465.74*	d, h
THBS1	299.96	*696.28*	**239.23**	*538.79*	*819.96*	
THBS2	45.80	*68.03*	*61.96*	*97.85*	*94.65*	
THBS3	33.11	**19.98**	**10.57**	**17.22**	**23.76**	
VCAN	98.11	*279.02*	*188.23*	*237.38*	*334.81*	a, c, d, h
HAS2	234.75	*307.97*	**100.67**	*401.46*	*505.58*	

Bold: reduction in expression, compared to 2D standard. Italic: increase in expression, compared to 2D standard. Underlined: FPKM < 1. a: 2D versus BSA; b: 2D versus 3D/FN; c: 2D versus 3D/Col1; d: 2D versus 3D/LN; e: BSA versus 3D/I; f: BSA versus 3D/LN; g: 3D/FN versus 3D/Col1; h: 3D/FN versus 3D/LN.

**Table 2:** Expression levels of integrins in hMSCs cultured for 28 days in 2D standard and 3D ECM-based structure conditions.

Gene	Average FPKM					Significance
	2D/D28	3D/BSA	3D/FN	3D/Col1	3D/LN	
ITGA1	1.28	*5.34*	*2.93*	*5.12*	*7.34*	
ITGA2	2.18	*9.54*	*4.37*	*4.18*	*11.33*	
ITGA3	1.40	0.55	0.31	0.72	0.39	
ITGA4	0.68	*15.22*	*3.15*	*2.55*	**0.63**	a
ITGA5	62.99	**36.35**	**24.16**	**25.63**	**55.23**	
ITGAE	19.68	**16.17**	**17.53**	**11.81**	**16.99**	
ITGAV	3.89	*17.92*	*13.74*	*33.58*	*18.13*	c, g
ITGA6	0.60	*2.49*	*7.93*	*3.76*	0.96	
ITGA7	0.15	*2.82*	0.23	0.18	**0.00**	
ITGA8	1.81	0.00	0.38	**1.74**	**1.79**	
ITGA10	3.98	*20.99*	*19.55*	*27.72*	*50.63*	d
ITGA11	33.87	**25.68**	**18.59**	*46.22*	*50.06*	
ITGB1	280.32	*547.67*	*456.69*	*572.81*	*551.84*	
ITGB2	0.16	0.00	0.59	*6.30*	0.00	
ITGB4	0.00	0.00	0.50	*2.84*	0.00	
ITGB5	37.11	**27.88**	**25.67**	**29.49**	**22.85**	
ITGB8	0.68	12.44	4.52	11.86	6.27	

Bold: reduction in expression, compared to 2D standard. Italic: increase in expression, compared to 2D standard. Underlined: FPKM < 1. a: 2D versus BSA; b: 2D versus 3D/FN; c: 2D versus 3D/Col1; d: 2D versus 3D/LN; e: BSA versus 3D/I; f: BSA versus 3D/LN; g: 3D/FN versus 3D/Col1; h: 3D/FN versus 3D/LN.

**Table 3:** Expression levels of MMPS, ADAMS, and TIMPs in hMSCs cultured for 28 days in 2D standard and 3D ECM-based structure conditions.

Gene	Average FPKM					Significance
	2D/D28	3D/BSA	3D/FN	3D/Col1	3D/LN	
MMP1	48.12	*822.48*	*283.69*	*640.14*	*1395.91*	d
MMP2	689.96	**598.60**	**601.51**	*830.16*	*1332.27*	
MMP3	8.08	*10.23*	**3.77**	**8.00**	*19.02*	
MMP8	0.00	*10.62*	0.26	0.00	*7.48*	
MMP11	5.08	**1.71**	**1.71**	0.00	0.00	
MMP13	9.76	*40.09*	*111.21*	*76.32*	*224.58*	d, f
MMP14	2.43	*3.87*	**1.02**	**1.41**	0.81	
MMP16	3.62	*4.69*	*4.40*	*26.89*	*6.98*	c, e, g
MMP19	14.86	**12.39**	**7.18**	*29.18*	**10.34**	
MMP24	1.65	0.45	0.37	**1.34**	0.17	
ADAM1	0.00	0.00	0.27	0.31	2.18	
ADAM9	29.28	*39.54*	*40.17*	*30.69*	*42.11*	
ADAM10	9.86	*15.55*	*15.02*	*22.07*	*49.41*	d, f, h
ADAM12	20.52	*67.73*	*26.70*	*46.95*	*49.82*	a, b
ADAM15	0.19	0.00	0.25	*2.97*	*1.08*	
ADAM17	7.86	*15.32*	*14.24*	*12.09*	*14.60*	
ADAM19	31.25	**6.38**	**3.71**	**6.91**	**4.20**	a, b, c, d
ADAM21	0.00	*1.72*	0.02	0.00	0.00	
ADAM23	2.55	0.39	0.18	**1.20**	*2.91*	
ADAM33	0.26	0.00	*2.07*	0.94	0.00	
TIMP1	18055.04	**2719.30**	**5834.20**	**2490.98**	**3835.62**	a, c, d
TIMP2	68.19	*160.04*	*127.25*	*143.54*	*154.66*	
TIMP3	1321.25	*2670.46*	**879.00**	*2115.14*	*3145.51*	h

Bold: reduction in expression, compared to 2D standard. Italic: increase in expression, compared to 2D standard. Underlined: FPKM < 1. a: 2D versus BSA; b: 2D versus 3D/FN; c: 2D versus 3D/Col1; d; 2D versus 3D/LN; e: BSA versus 3D/I; f: BSA versus 3D/LN; g: D/FN versus 3D/Col1; h: 3D/FN versus 3D/LN.

## Abbreviations

ECM: Extracellular matrix
hMSC: Human mesenchymal stem cells
PEG: Polyethylene glycol
FN: Fibronectin
COLI: Type I collagen
LN: Laminin-111
FPKM: Fragments per kilobase pair per million mapped reads
COL1A1: Alpha 1 chain of type I collagen
SGPL1: Sphingosine-1-phosphate lyase 1
MAGED2: MAGE family member D2
GO: Gene ontology
MMP: Matrix metalloproteinase
VCAM: Vascular cell adhesion molecule
MCAM: Melanoma cell adhesion molecule
ACTA2: Smooth muscle aortic alpha-actin
TAGLN: Transgelin
mESC: Mouse embryonic stem cell
MEK: Mitogen-activated protein kinase kinase
ERK: Extracellular signal-regulated kinase
FPGA: Field-programmable gate array
FIFO: First-in, first-out
PMT: Photomultiplier tube
GUI: Graphical user interface
BSA: Bovine serum albumin
OTDS: Octadecyltrichlorosilane
FLIM: Fluorescence lifetime imaging microscopy
NA: Numerical aperture
SEM: Scanning electron microscopy
SCDE: Single-cell differential expression
DAVID: Database for Annotation, Visualization and Integrated Discovery
ANOVA: Analysis of variance.

## Supplementary Materials

Supplementary 1 Supplemental Table 1: contains raw data related to the tracking of physical structure modification.

Supplementary 2 Supplemental Table 2: contains FPKM data for all transcripts of all samples.

Supplementary 3 Supplemental Table 3: contains differentially expressed genes between sample types.

Supplementary 4 Supplemental Table 4: contains gene ontology analyses.

## References

[1] Linsley C., Wu B., Tawil B. (2013). The effect of fibrinogen, collagen type I, and fibronectin on mesenchymal stem cell growth and differentiation into osteoblasts. *Tissue Engineering. Part A*.

[2] Lu H., Hoshiba T., Kawazoe N., Koda I., Song M., Chen G. (2011). Cultured cell-derived extracellular matrix scaffolds for tissue engineering. *Biomaterials*.

[3] Cukierman E., Pankov R., Yamada K. M. (2002). Cell interactions with three-dimensional matrices. *Current Opinion in Cell Biology*.

[4] Stegemann J. P., Nerem R. M. (2003). Altered response of vascular smooth muscle cells to exogenous biochemical stimulation in two- and three-dimensional culture. *Experimental Cell Research*.

[5] Jung J. P., Sprangers A. J., Byce J. R. (2013). ECM-incorporated hydrogels cross-linked via native chemical ligation to engineer stem cell microenvironments. *Biomacromolecules*.

[6] Jung J. P., Bache-Wiig M. K., Provenzano P. P., Ogle B. M. (2016). Heterogeneous differentiation of human mesenchymal stem cells in 3D extracellular matrix composites. *BioResearch Open Access*.

[7] Becerra-Bayona S., Guiza-Arguello V., Qu X., Munoz-Pinto D. J., Hahn M. S. (2012). Influence of select extracellular matrix proteins on mesenchymal stem cell osteogenic commitment in three-dimensional contexts. *Acta Biomaterialia*.

[8] Jung J. P., Hu D., Domian I. J., Ogle B. M. (2016). An integrated statistical model for enhanced murine cardiomyocyte differentiation via optimized engagement of 3D extracellular matrices. *Scientific Reports*.

[9] Astrof S., Crowley D., Hynes R. O. (2007). Multiple cardiovascular defects caused by the absence of alternatively spliced segments of fibronectin. *Developmental Biology*.

[10] George E. L., Georges-Labouesse E. N., Patel-King R. S., Rayburn H., Hynes R. O. (1993). Defects in mesoderm, neural tube and vascular development in mouse embryos lacking fibronectin. *Development*.

[11] Costa-Silva B., da Costa M. C., Melo F. R. (2009). Fibronectin promotes differentiation of neural crest progenitors endowed with smooth muscle cell potential. *Experimental Cell Research*.

[12] Mummery C. L., Zhang J., Ng E. S., Elliott D. A., Elefanty A. G., Kamp T. J. (2012). Differentiation of human embryonic stem cells and induced pluripotent stem cells to cardiomyocytes: a methods overview. *Circulation Research*.

[13] Abolpour Mofrad S., Kuenzel K., Friedrich O., Gilbert D. F. (2016). Optimizing neuronal differentiation of human pluripotent NT2 stem cells in monolayer cultures. *Development, Growth & Differentiation*.

[14] Zhang X., Bendeck M. P., Simmons C. A., Santerre J. P. (2017). Deriving vascular smooth muscle cells from mesenchymal stromal cells: evolving differentiation strategies and current understanding of their mechanisms. *Biomaterials*.

[15] Lu P., Takai K., Weaver V. M., Werb Z. (2011). Extracellular matrix degradation and remodeling in development and disease. *Cold Spring Harbor Perspectives in Biology*.

[16] Davis G. E., Senger D. R. (2005). Endothelial extracellular matrix: biosynthesis, remodeling, and functions during vascular morphogenesis and neovessel stabilization. *Circulation Research*.

[17] Cunningham L. P., Veilleux M. P., Campagnola P. J. (2006). Freeform multiphoton excited microfabrication for biological applications using a rapid prototyping CAD-based approach. *Optics Express*.

[18] Ajeti V., Lien C. H., Chen S. J. (2013). Image-inspired 3D multiphoton excited fabrication of extracellular matrix structures by modulated raster scanning. *Optics Express*.

[19] Su P. J., Tran Q. A., Fong J. J., Eliceiri K. W., Ogle B. M., Campagnola P. J. (2012). Mesenchymal stem cell interactions with 3D ECM modules fabricated via multiphoton excited photochemistry. *Biomacromolecules*.

[20] Balasubramanian D., Du X., Zigler J. S. (1990). The reaction of singlet oxygen with proteins, with special reference to crystallins. *Photochemistry and Photobiology*.

[21] Trivedi P., Hematti P. (2007). Simultaneous generation of CD34^+^ primitive hematopoietic cells and CD73^+^ mesenchymal stem cells from human embryonic stem cells cocultured with murine OP9 stromal cells. *Experimental Hematology*.

[22] Trivedi P., Hematti P. (2008). Derivation and immunological characterization of mesenchymal stromal cells from human embryonic stem cells. *Experimental Hematology*.

[23] Kharchenko P. V., Silberstein L., Scadden D. T. (2014). Bayesian approach to single-cell differential expression analysis. *Nature Methods*.

[24] Freeman B. T., Jung J. P., Ogle B. M. (2016). Single-cell RNA-seq reveals activation of unique gene groups as a consequence of stem cell-parenchymal cell fusion. *Scientific Reports*.

[25] Santiago J. A., Pogemiller R., Ogle B. M. (2009). Heterogeneous differentiation of human mesenchymal stem cells in response to extended culture in extracellular matrices. *Tissue Engineering. Part A*.

[26] Zeltz C., Lu N., Gullberg D. (2014). Integrin *α*11*β*1: a major collagen receptor on fibroblastic cells. *Advances in Experimental Medicine and Biology*.

[27] Zhang X., Groopman J. E., Wang J. F. (2005). Extracellular matrix regulates endothelial functions through interaction of VEGFR-3 and integrin *α*
_5_
*β*
_1_. *Journal of Cellular Physiology*.

[28] Torzilli P. A., Bhargava M., Chen C. T. (2011). Mechanical loading of articular cartilage reduces IL-1-induced enzyme expression. *Cartilage*.

[29] Pardo A., Selman M. (2005). MMP-1: the elder of the family. *The International Journal of Biochemistry & Cell Biology*.

[30] Birkedal-Hansen H., Moore W. G., Bodden M. K. (1993). Matrix metalloproteinases: a review. *Critical Reviews in Oral Biology & Medicine*.

[31] Chandler S., Miller K. M., Clements J. M. (1997). Matrix metalloproteinases, tumor necrosis factor and multiple sclerosis: an overview. *Journal of Neuroimmunology*.

[32] Kahari V. M., Saarialho-Kere U. (1997). Matrix metalloproteinases in skin. *Experimental Dermatology*.

[33] Binder B. Y., Williams P. A., Silva E. A., Leach J. K. (2015). Lysophosphatidic acid and sphingosine-1-phosphate: a concise review of biological function and applications for tissue engineering. *Tissue Engineering. Part B, Reviews*.

[34] Skoura A., Sanchez T., Claffey K., Mandala S. M., Proia R. L., Hla T. (2007). Essential role of sphingosine 1-phosphate receptor 2 in pathological angiogenesis of the mouse retina. *The Journal of Clinical Investigation*.

[35] Kono M., Mi Y., Liu Y. (2004). The sphingosine-1-phosphate receptors S1P_1_, S1P_2_, and S1P_3_ function coordinately during embryonic angiogenesis. *The Journal of Biological Chemistry*.

[36] Wijelath E. S., Rahman S., Murray J., Patel Y., Savidge G., Sobel M. (2004). Fibronectin promotes VEGF-induced CD34^+^ cell differentiation into endothelial cells. *Journal of Vascular Surgery*.

[37] Battista S., Guarnieri D., Borselli C. (2005). The effect of matrix composition of 3D constructs on embryonic stem cell differentiation. *Biomaterials*.

[38] Clark R. A., DellaPelle P., Manseau E., Lanigan J. M., Dvorak H. F., Colvin R. B. (1982). Blood vessel fibronectin increases in conjunction with endothelial cell proliferation and capillary ingrowth during wound healing. *The Journal of Investigative Dermatology*.

[39] Saidak Z., Le Henaff C., Azzi S. (2015). Wnt/*β*-catenin signaling mediates osteoblast differentiation triggered by peptide-induced *α*5*β*1 integrin priming in mesenchymal skeletal cells. *The Journal of Biological Chemistry*.

[40] Dzobo K., Vogelsang M., Parker M. I. (2015). Wnt/*β*-catenin and MEK-ERK signaling are required for fibroblast-derived extracellular matrix-mediated endoderm differentiation of embryonic stem cells. *Stem Cell Reviews and Reports*.

